# Modelling the Influence of Different Soot Types on the Radio-Frequency-Based Load Detection of Gasoline Particulate Filters

**DOI:** 10.3390/s20092659

**Published:** 2020-05-06

**Authors:** Stefanie Walter, Peter Schwanzer, Gunter Hagen, Gerhard Haft, Hans-Peter Rabl, Markus Dietrich, Ralf Moos

**Affiliations:** 1 Bayreuth Engine Research Center (BERC), Department of Functional Materials, University of Bayreuth, 95447 Bayreuth, Germany; functional.materials@uni-bayreuth.de (S.W.); functional.materials@uni-bayreuth.de (G.H.); 2 Combustion Engines and Emission Control Laboratory (CEEC), Ostbayerische Technische Hochschule Regensburg, 93053 Regensburg, Germany; Peter.Schwanzer@oth-regensburg.de (P.S.); Hans-Peter.Rabl@oth-regensburg.de (H.-P.R.); 3 Vitesco Technologies GmbH, 93055 Regensburg, Germany; Gerhard.Haft@continental-corporation.com (G.H.); Markus.Dietrich@continental-corporation.com (M.D.)

**Keywords:** gasoline particulate filter (GPF), radio frequency (RF), soot mass determination, finite element method (FEM), ash, diesel particulate filter (DPF)

## Abstract

Gasoline particulate filters (GPFs) are an appropriate means to meet today’s emission standards. As for diesel applications, GPFs can be monitored via differential pressure sensors or using a radio-frequency approach (RF sensor). Due to largely differing soot properties and engine operating modes of gasoline compared to diesel engines (e.g., the possibility of incomplete regenerations), the behavior of both sensor systems must be investigated in detail. For this purpose, extensive measurements on engine test benches are usually required. To simplify the sensor development, a simulation model was developed using COMSOL Multiphysics^®^ that not only allowed for calculating the loading and regeneration process of GPFs under different engine operating conditions but also determined the impact on both sensor systems. To simulate the regeneration behavior of gasoline soot accurately, an oxidation model was developed. To identify the influence of different engine operating points on the sensor behavior, various samples generated at an engine test bench were examined regarding their kinetic parameters using thermogravimetric analysis. Thus, this compared the accuracy of soot mass determination using the RF sensor with the differential pressure method. By simulating a typical driving condition with incomplete regenerations, the effects of the soot kinetics on sensor accuracy was demonstrated exemplarily. Thereby, the RF sensor showed an overall smaller mass determination error, as well as a lower dependence on the soot kinetics.

## 1. Introduction

In recent years, the public focus regarding the emissions of harmful automotive pollutants has primarily been on diesel vehicles and their high raw emissions of nitrogen oxides compared to gasoline engines. However, with the tightening of exhaust emission standards, even gasoline engines require more complex exhaust after-treatment systems. In particular, gasoline direct injection (GDI) engines emit a large number of soot particles compared with port-fuel-injected engines due to the inhomogeneous mixture of fuel and intake air in the combustion chamber. Due to the smaller soot size compared to diesel particles, exhaust emission standards, which only limit the particulate mass, can be met solely using engine-based means. With the introduction of a limited particulate number as part of the Euro 6b standard and the real driving emissions test procedure (Euro 6d-Temp), gasoline particulate filters (GPF) have become necessary [1,2].

Similar to diesel particulate filters (DPFs), GPFs are based on the wall-flow filter principle [3]. However, gasoline engines have very different operating conditions, such as higher exhaust gas temperatures and exhaust mass flows. Therefore, adjustments are required for the application of GPFs (e.g., regarding the mean pore size of the filter walls) [4]. Additionally, the different operating conditions affect filter monitoring concerning soot loading and failure detection. The load detection of diesel particulate filters is performed according to state-of-the-art methods using a model based on the differential pressure (Δ*p*) between up- and downstream of the filter [5]. This system also has to be examined for application in GPFs. In addition to the direct influence of exhaust gas on the backpressure, the properties of soot particles differ and could cause a different sensor behavior. For instance, gasoline soot shows a higher reactivity during combustion with oxygen [6]. In combination with higher exhaust gas temperatures, this may lead to passive regenerations during fuel cuts [4,7]. In the case of incomplete regenerations, which may occur due to the termination of fuel cuts or due to short switches to lean engine operation modes combined with too low temperatures in certain areas of the filter, a lower differential pressure than expected may occur as a result of a changed flow distribution in the filter [8,9]. Furthermore, in a clean filter, soot particles are first deposited in the filter walls and cause a higher backpressure increase than in the subsequent soot cake formation [10,11]. In contrast, during regeneration, oxidation takes place regardless of the storage location. This may result in the presence of a soot cake, while the soot in the deep bed has already burnt off, which can be associated with a change in the backpressure characteristics [12]. 

A possible method for monitoring GPFs more precisely can be realized using soot concentration sensors based on electrostatic or thermophoretic principles [13,14]. They can be used not only for on-board diagnostic purposes but also to determine the load in the filter via the integration of their signals. Instead of determining the soot load indirectly, a radio-frequency approach (RF sensor) can be used to directly detect the amount of soot stored via its dielectric properties. The RF sensor uses the metallic filter canning as the conductive boundary of a cavity resonator. Coaxial probe antennas produce electromagnetic waves, whereby their propagation is influenced by the dielectric properties of the filter or catalyst inside the canning. This influence can be measured by evaluating the resonant frequencies $f_{\mathrm{res}}$ or quality factors $Q_{0}$ of certain resonant modes [15,16]. Although the resonant modes at higher soot loads cannot be evaluated due to the high conductivity of soot, a load determination can be achieved using the transmission losses averaged over a certain frequency range [17]. With DPFs, the functionality of this sensor approach has already been demonstrated [17,18,19,20]. The first investigations of GPFs also showed the general ability to detect soot masses [21]. Besides filter monitoring, the RF sensor is also suitable for catalyst state determination [22]. Thus, the ammonia loading of selective catalytic reduction (SCR) catalysts or the oxygen storage of three-way catalytic converters (TWCs) can be measured [16,23,24,25,26]. Even the simultaneous evaluation of two different parameters is possible, as can be shown with the nitrogen oxide and the oxygen storage of a lean NO_x_ trap (LNT) [27,28]. Thus, it would be possible to monitor GPFs with a TWC coating not only for soot loading but also concurrently for oxygen storage [21].

However, even with the RF sensor, the knowledge acquired from DPFs cannot be directly adopted. Gasoline soot particles differ not only in their size from diesel particles but also in their nanostructure [29]. Furthermore, the selected engine operating point and the combusted fuel affect the kinetic properties of the soot oxidation [30]. Therefore, time-consuming and cost-intensive engine measurements are necessary for the serial use of both sensor systems. To simplify the adaptation of the RF sensor as well as the differential pressure sensor to the gasoline particulate filter, a simulation model was developed with the simulation software COMSOL Multiphysics^®^ 5.4 (COMSOL Inc., Stockholm, Sweden) to determine the signals of both sensor systems under different operating conditions. Thereby, the simulation model should not simulate all processes perfectly realistically, but rather help to recognize possible perturbations to the sensor signals. Thus, the here-given simulation model can help to develop methods to eliminate these influences during the processing of the measured signal. As an example, this study showed the effects of the different reaction kinetics of various soot types on the sensor accuracy during incomplete regenerations. Therefore, this simulation model allows for a direct comparison between the accuracy of the two sensor concepts under exactly defined operating conditions.

## 2. Simulation Model Design

**Figure 1:**
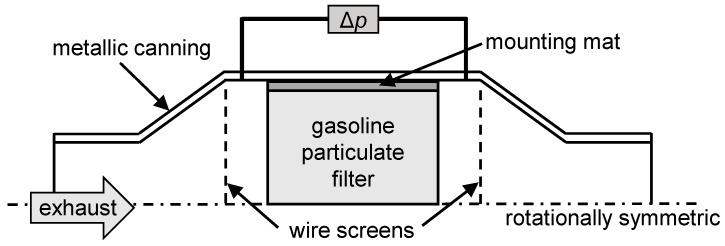
Schematic layout of the simulation model. The wire screens are used to exactly define the cavity resonator in the radio-frequency (RF) simulation.

**Figure 2:**
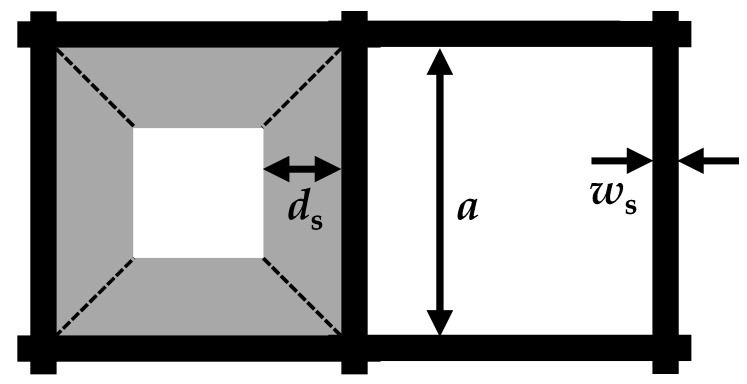
Schematic structure of an inlet (left) and an outlet filter channel (right) with width a, wall thickness $w_{s}$, and soot cake thickness in the inlet channel $d_{s}$ (adapted from Konstandopoulos et al. [32]).

To enable an exact determination of the sensor behavior combined with a short computing time, the model was split into two parts. A similar approach was used in Dietrich et al. [31] to describe the radio frequency signal for reactions in an SCR catalyst. In the first step, the time-dependent soot loading is computed. For this purpose, the model considers not only the flow conditions in the exhaust gas system but also the resulting temperature distribution and reaction kinetics of the soot stored in the GPF. Additionally, the differential pressure signal is determined in this model. Due to the immense computational effort that would be associated with detailed modeling of the GPF including flow channels, the filter was regarded as a homogeneous, porous medium. This allowed for the model to be simplified further to a rotationally symmetrical geometry (schematically shown in Figure 1). Effects such as the influence of soot loading on the permeability of the filter walls or a changing channel cross-section during soot cake formation are described using analytical equations. Therefore, a monolithic filter with squared channels was assumed (Figure 2). The formation of a soot cake occurs uniformly on all four side-walls of the inlet channels. In addition to the formation of a soot cake, the soot particles can also be deposited in the pore structure of the filter walls. This is also known as “deep bed deposition.” 

The RF signal is determined in a second step using a three-dimensional resonator model, in which the filter is also considered homogeneous. To calculate the time-dependent RF signal, not only the soot loading but also the temperature distribution is transferred from the first model since this has a considerable influence on the dielectric properties of soot. The resonator geometry is defined by the area between the wire screens shown in Figure 1. However, in the flow simulation, these grids are not considered. The geometry of the simulation model is based on a real setup on an engine test bench. The differential pressure measurement takes place between two points within the widening of the canning to the filter diameter shortly before and after the GPF (Figure 1). To compute the flow model, a *Segregated Solver* partitions the variables to be calculated into several groups, which are solved separately using the direct *PARDISO* solver, whereas the computation of the RF model is performed using the direct *MUMPS* solver [33].

### 2.1. Flow Distribution in GPFs

To calculate the exhaust gas flow, the simulation model is divided into two areas, i.e., an inlet and an outlet part. Both are coupled in the area of the GPF via Darcy’s law. It describes the relationship between the flow velocity through a porous medium and the resulting backpressure [34]. In this way, the local mass flow ${\dot{m}}_{\mathrm{exhaust}}$ through the filter walls between the inlet and outlet channels related to the filter volume can be calculated as a function of the pressure difference Δ*p* using Equation (1):

(1) $$\dot{m}=\frac{1}{2}\rho \Delta p\cdot \alpha_{\mathrm{filter}}.$$

The relationship depends not only on the density of the exhaust gas *ρ* but also on the factor $\alpha_{\mathrm{filter}}$. The latter is termed in this study as the penetrability of the filter and, in the case of a clean filter, depends on the geometry of the filter channels, the dynamic viscosity of the exhaust gas *η*, and the permeability of the filter walls $\kappa_{0}$:

(2) $$\alpha_{\mathrm{filter}}=\alpha_{\mathrm{clean}}=\frac{{4\kappa }_{0}a}{{\eta w}_{s}{\left(a+w_{s}\right)}^{2}}.$$

However, in the case of a soot-loaded filter, additional flow resistance caused by deep bed filtration and the soot cake must be considered. Therefore, the reciprocal values of the individual penetrabilities must be summed up:

(3) $$\alpha_{\mathrm{filter}}={\left(\frac{1}{\alpha_{\mathrm{clean}}}+\frac{1}{\alpha_{\mathrm{cake}}}+\frac{1}{\alpha_{\mathrm{deep}}}\right)}^{-1}.$$

Likewise, the description of the soot cake penetrability $\alpha_{\mathrm{cake}}$ is also based on Darcy’s law. However, it must be considered that the width of the soot layer decreases trapezoidally with the soot cake thickness (Figure 2). Assuming a constant soot permeability $\kappa_{s}$, this results in a non-linear relationship with the soot layer thickness $d_{s}$ due to the changing flow cross-section of the soot cake [32]:

(4) $$\alpha_{\mathrm{cake}}=\frac{\kappa_{s}}{\eta }\frac{16}{{\left(a+w_{s}\right)}^{2}}{\left(\ln\frac{a}{a-{2d}_{s}}\right)}^{-1}.$$

The deposition of soot in the deep bed also changes the permeability of the filter walls. In this model, this is not accomplished by varying the permeability of the filter wall, but instead by introducing a further penetrability $\alpha_{\mathrm{deep}}$ (Equation (5)). To determine this, a virtual soot thickness $d_{s,\mathrm{deep}}$ of the deep bed is introduced, which is calculated using the soot mass stored there relative to the filter surface and the apparent density of a soot layer. For a correct description of the backpressure, the three permeabilities must be determined experimentally.

(5) $$\alpha_{\mathrm{deep}}=\frac{{4\kappa }_{s,\mathrm{deep}}a}{{\eta d}_{s,\mathrm{deep}}{\left(a+w_{s}\right)}^{2}}$$

In addition to the filter penetrability, the flow in the areas before and after the GPF and in the channels must be considered for a correct determination of the backpressure. Due to high exhaust mass flows in gasoline engines, which are specified in the model as a boundary condition, laminar flow cannot be assumed in areas outside the GPF. Therefore, the algebraic yPlus turbulence model was used for a proper exhaust flow calculation. In contrast, in the filter channels, laminar flow was assumed owing to their small cross-sections [35,36]. The backpressure caused by channel friction can be calculated using the Hagen–Poiseuille equation [36,37]. Since in the simulation model, the filter is seen as a porous medium, the channel friction can be modeled using Darcy’s law as a form of permeability in the flow direction (the factor *F* is equal to 28.454 for a fully developed laminar flow):

(6) $$\kappa_{\mathrm{channel}}=\frac{1}{2F}\frac{{\left(a-{2d}_{s}\right)}^{4}}{{\left(a+w_{s}\right)}^{2}}.$$

However, recent investigations have shown that due to the high mass flows in GPFs turbulent friction, losses can also occur in the channels [38]. These observations have not been considered in this study but will be taken into account in the further development of the simulation model.

### 2.2. Determination of the Filter Temperature

In addition to the flow conditions, a precise description of the soot distribution in the GPF also requires knowledge about the temperature in the exhaust system. Besides the influence on the flow behavior, the temperature affects the oxidation rate during regeneration. As in the flow calculation, the inlet and outlet sides are treated separately. Additionally, the temperature of the filter material $T_{\mathrm{GPF}}$ and the thermal mass of the filter themselves are considered as well. 

The sections are coupled via two different types of heat transfer. First, the mass flow through the filter causes a heat flow between the inlet channels and the filter walls. This, in turn, transmits the heat, together with the exhaust gas, to the outlet channels. The heat flow into the GPF ${\dot{Q}}_{\mathrm{GPF}}$ can be determined using Equation (7) by knowing the mass flow distribution, the heat capacity of the exhaust gas $c_{p,\mathrm{exhaust}}$, and the temperature difference to the filter channels ($T_{\mathrm{inlet}}$ and $T_{\mathrm{outlet}}$ are the temperatures in the inlet and outlet channels, respectively):

(7) $${\dot{Q}}_{\mathrm{GPF}}=\dot{m}c_{p,\mathrm{exhaust}}\left(T_{\mathrm{inlet}/\mathrm{outlet}}-T_{\mathrm{GPF}}\right).$$

Additionally, heat exchange between the channels and the GPF also occurs due to convection caused by exhaust gas flowing past the filter channel walls. The heat transfer coefficients for these are calculated using the equations from References [39,40]. The exhaust gas system is not a thermally isolated system; instead, it emits heat to ambient air and thus reduces the GPF temperature at the outer region. In the model, this is implemented by considering heat conduction through the metallic canning walls and heat convection at their outer walls. The exhaust gas system is simplified as a long horizontal cylinder through which natural convection, which represents a stationary vehicle, takes place. Additional cooling by forced convection, which would cause airflow, has not been considered in this study. The equations used to determine the heat transfer coefficients can be found in the Heat Transfer Module User’s Guide [41]. Due to occasionally high exhaust gas temperatures, heat radiation according to the Stefan–Boltzmann law is also taken into account. During regeneration, the filter material is additionally heated up by the exothermic regeneration reaction. All required enthalpies were taken from McBride et al. [42].

### 2.3. Soot Storage

Based on the exhaust gas flow through the GPF, the location of the soot deposit can be determined. Due to the low mass of the soot particles, it can be assumed that they follow the exhaust gas flow in the filter channels [43]. Based on the incoming soot mass ${\dot{m}}_{\mathrm{soot},\mathrm{in}}$ and the volume flow into the system ${\dot{V}}_{\mathrm{exhaust}}$, a homogeneous soot concentration $c_{\mathrm{soot}}$ for the inlet side of the filter is calculated (Equation (8)). Combined with the mass flow distribution, this leads to a soot mass rate flowing through the filter walls ${\dot{m}}_{\mathrm{soot},\mathrm{exhaust}}$ (Equation (9)) [44]:

(8) $$c_{\mathrm{soot}}=\frac{{\dot{m}}_{\mathrm{soot},\mathrm{in}}}{{\dot{V}}_{\mathrm{exhaust}}},$$

(9) $${\dot{m}}_{\mathrm{soot},\mathrm{exhaust}}=\frac{c_{\mathrm{soot}}\cdot {\dot{m}}_{\mathrm{exhaust}}}{\rho }.$$

However, owing to the highly porous filter walls, not all of the mass flow ${\dot{m}}_{\mathrm{soot},\mathrm{exhaust}}$ is deposited in the filter. At the beginning of loading a clean filter, soot is deposited in the filter pores, the so-called deep bed. The filtration efficiency of this layer can be described by assuming it is made of so-called unit collectors [36]. This model is used in a large number of filtration models [3,44,45,46,47,48]. The filtration efficiency depends on the amount of soot stored in the deep bed. Using the equations in Konstandopoulos and Johnson [36], a length-related filtration efficiency can be calculated. Since the deposition rate is not the same across the entire filter wall due to a decrease in the soot concentration as a result of filtration, the filtration efficiency also has a location dependency. To determine a correct deep bed filtration efficiency $\eta_{\mathrm{bed}}$, even during regeneration, numerical calculations using a separate three-dimensional model of the filter wall coupled with the main model is used. Additionally, this model is also used to calculate the soot fraction ϕ that does not deposit in the deep bed but instead starts forming a soot cake [32]. The formed soot cake in the filter channel retroactively influences the storage rate in the deep bed due to its filtration efficiency $\eta_{\mathrm{cake}}$. This is calculated using the geometric coverage by a soot particle layer based on the bulk density of soot and the apparent density of the soot cake. Based on the filtration efficiencies $\eta_{\mathrm{cake}}$ and $\eta_{\mathrm{bed}}$, as well as ϕ, the storage rates of the soot cake ${\dot{m}}_{\mathrm{soot},\mathrm{cake}}$ (Equation (10)) and the deep bed ${\dot{m}}_{\mathrm{soot},\mathrm{bed}}$ (Equation (11)) can now be calculated. Using time integration, both soot loads can be determined:

(10) $${\dot{m}}_{\mathrm{soot},\mathrm{cake}}=\left(\eta_{\mathrm{cake}}+\phi \left(1-\eta_{\mathrm{cake}}\right)\right){\dot{m}}_{\mathrm{soot},\mathrm{exhaust}},$$

(11) $${\dot{m}}_{\mathrm{soot},\mathrm{bed}}=\eta_{\mathrm{bed}}\left(1-\eta_{\mathrm{cake}}\right)\left(1-\phi \right)\;{\dot{m}}_{\mathrm{soot},\mathrm{exhaust}}.$$

### 2.4. Reaction Kinetics of Soot

In addition to the storage behavior, the kinetics of soot oxidation is very important for the description of the GPF loading. Particularly when considering incomplete regenerations, the kinetics has a strong influence on the local soot distribution, as well as on the stored amount in both the soot cake and the deep bed. The model considers the oxidation of carbon to CO_2_, as well as the incomplete oxidation to CO (Equations (12) and (13)):

(12) $${C+O}_{2}\;\to \;{\mathrm{CO}}_{2},$$

(13) $$C+\frac{1}{2}O_{2}\;\to \;\mathrm{CO}.$$

However, it is not necessary to consider the kinetics of both reactions separately. According to References [49,50], the ratio of emitted CO_2_ to CO can be expressed using a factor $f_{\mathrm{CO}}$ that depends on the temperature and oxygen content in the exhaust gas (Equation (14)):

(14) $$f_{\mathrm{CO}}\left(T_{\mathrm{GPF}}{,c}_{O_{2}}\right)=\frac{c_{\mathrm{CO}}}{c_{\mathrm{CO}}+c_{{\mathrm{CO}}_{2}}}.$$

Nevertheless, this relationship only influences the carbon combustion rate indirectly by affecting the oxygen available in the filter walls. The reaction rate of the carbon ${\dot{m}}_{\mathrm{soot}}$ can be described independently of $f_{\mathrm{CO}}$ using Equation (15) via a first-order reaction with respect to the oxygen concentration $c_{O_{2}}$ and *n* for the reaction order of the remaining soot mass [51,52]:

(15) $${\dot{m}}_{\mathrm{soot}}=-m_{\mathrm{soot}}^{n}c_{O_{2}}M_{c}k_{c}(T_{\mathrm{GPF}})\;\mathrm{with}\;k_{c}=k_{c,0}\cdot \exp\left(-\frac{E_{A}}{R\cdot T_{\mathrm{GPF}}}\right).$$

The temperature dependence of the combustion is determined by the pre-exponential factor $k_{c,0}$ and the activation energy $E_{A}$. For comparability with literature values, the molar mass of carbon $M_{c}=12$ g/mol is also included, such that the unit of $k_{c,0}$ is m^3^/kg·s [52]. For simplicity, the simulations and the corresponding reaction kinetic analyses assume a first-order reaction similar to several models in the literature [50,53]. An implementation of reaction orders different from one would be possible for complete regenerations. On the other hand, in the case of incomplete oxidation and a subsequent loading, a description of the effects by mixed partially regenerated and fresh soot on the reaction order would be necessary. In simulation models for diesel engines, NO_2_ combustion is also taken into account in some cases [50]. Due to the stoichiometric operation of gasoline engines and the resulting lower number of nitrogen oxides after the TWC, this model does not include that reaction.

Based on the conversion rate of oxygen, which depends on the position in the soot layer and the gas velocity $v_{w}$ through the filter walls, it is possible to describe the reaction rate during filter regeneration (and not just for constant oxygen concentrations) [32]. This results in an exponential relationship for the decrease of the total amount of soot in the filter $m_{\mathrm{soot}}$ (Equation (16)). However, the combustion in the deep bed and the soot cake does not take place at the same rate because parts of the oxygen are already consumed while flowing through the soot cake. This effect is taken into account by calculating the local oxygen distribution. Thus, loading and regeneration processes can be described separately for the soot cake and the deep filtration.

(16) $${\dot{m}}_{\mathrm{soot}}=\frac{12\frac{g}{\mathrm{mol}}}{1-\frac{f_{\mathrm{CO}}}{2}}c_{O_{2}}v_{w}\left[1-\exp\left(-\left(1-\frac{f_{\mathrm{CO}}}{2}\right)k_{c}\frac{m_{\mathrm{soot}}}{v_{w}\cdot 12\;\frac{g}{\mathrm{mol}}}\right)\right]$$

### 2.5. RF Parameter Calculation

The RF-parameters are calculated afterward using a modal analysis within a separate model that has the same geometry as the soot loading model [54]. The propagation of the electromagnetic waves can take place in the area between the grids and the interior walls of the metallic canning (Figure 1), where an impedance boundary condition with an electrical conductivity of 4.032 × 10^6^ S/m has been assigned to them. The GPF temperature and soot loading are transferred from the soot storage model to the RF-model, whereby no distinction is made between the deep bed and the soot cake. With these two parameters, the location-dependent complex dielectric parameters of the filter $\varepsilon_{\mathrm{filter}}^{\prime }$ and $\varepsilon_{\mathrm{filter}}^{\prime \prime }$ (which correspond to the real and imaginary part, respectively, of a complex relative permittivity $\varepsilon =\varepsilon^{\prime }-j\varepsilon^{\prime \prime }$), averaged over filter substrate, soot, and air, are calculated.

For this purpose, the dielectric properties of the filter substrate and the soot were determined by using the “microwave cavity perturbation” method described in Dietrich et al. [55] with a frequency of about 1.18 GHz, which is comparable to the lowest resonant mode considered in the simulation model (TE_111_). To help with modeling the influence of the temperature distribution in the GPF, the dielectric parameters are measured in a wide temperature range from room temperature to 600 °C. Due to too small amounts of real gasoline soot available, it was not possible to determine their permittivity using this method. For this reason, the dielectric properties of PrintexU were used in the simulations regardless of the type of soot. Since the gasoline soot used for the following investigations was deposited on cordierite substrates and the soot kinetics were determined using soot–cordierite mixtures, cordierite was applied as the substrate material in the simulation model.

**Figure 3:**
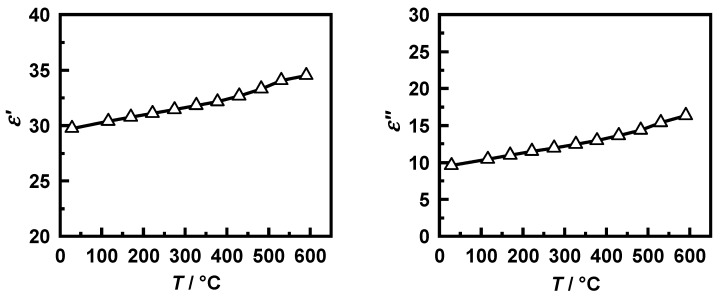
Measured dielectric parameters of PrintexU (left: $\varepsilon_{\mathrm{soot}}^{\prime }$; right: $\varepsilon_{\mathrm{soot}}^{\prime \prime }$) from 20 °C to 600 °C.

PrintexU shows a continuous increase in permittivity with temperature (Figure 3). For the implementation of the parameters in the simulation model, a linear regression was performed using the least-squares method. This resulted in the dependencies in Equations (17) and (18) with a determination coefficient *R*^2^ that was above 0.98 for both linear approximations. On the other hand, for cordierite, almost constant dielectric parameters were measured over the entire temperature range ($\varepsilon_{\mathrm{substrate}}^{\prime }=7.25$ and $\varepsilon_{\mathrm{substrate}}^{\prime \prime }=0.0009$).

(17) $$\varepsilon_{\mathrm{soot}}^{\prime }=29.26+0.00845\frac{1}{{}^{{}^{\circ}}C}\cdot T_{\mathrm{GPF}}$$

(18) $$\varepsilon_{\mathrm{soot}}^{\prime \prime }=8.94+0.0116\frac{1}{{}^{{}^{\circ}}C}\cdot T_{\mathrm{GPF}}$$

The mean dielectric properties of the soot-loaded filter are then found using Equation (19) (where the degree of polarization $\chi_{e}=\varepsilon^{\prime }-1$ is considered instead of $\varepsilon^{\prime }$) and Equation (20). The proportions of both components result from the density present in the filter relative to the bulk density. The latter was determined using a helium pycnometer Micromeritics AccuPyc 1330 (Micromeritics Instrument Corp., Norcross, GA, USA). For cordierite (obtained from a crushed GPF), the density was $\rho_{\mathrm{substrate}}$ = 2500 kg/m^3^, and for PrintexU, the density was $\rho_{\mathrm{soot}}$ = 1060 kg/m^3^. The density of the filter $\rho_{\mathrm{GPF}}$ was calculated using the mass and volume of the filter.

(19) $$\varepsilon_{\mathrm{filter}}^{\prime }-1=\frac{\rho_{\mathrm{GPF}}}{\rho_{\mathrm{substrate}}}{(\varepsilon }_{\mathrm{substrate}}^{\prime }-1)+\frac{m_{\mathrm{soot}}}{\rho_{\mathrm{soot}}}{(\varepsilon }_{\mathrm{soot}}^{\prime }-1)$$

(20) $$\varepsilon_{\mathrm{filter}}^{\prime \prime }=\frac{\rho_{\mathrm{GPF}}}{\rho_{\mathrm{substrate}}}\varepsilon_{\mathrm{substrate}}^{\prime \prime }+\frac{m_{\mathrm{soot}}}{\rho_{\mathrm{soot}}}\varepsilon_{\mathrm{soot}}^{\prime \prime }$$

Besides the filter, the dielectric parameters of air ($\varepsilon^{\prime }=1$, $\varepsilon^{\prime \prime }=0$, μ=1, and σ=0 S/m) were assigned to all other volumes in the RF model [31]. For the modal analysis, the resonant frequencies $f_{\mathrm{res}}$ and the quality factors $Q_{0}$ that correspond to the signals of the RF-sensor could then be determined directly by solving an eigenvalue problem [54]. However, when discussing the simulation results, the inverse quality factor $Q_{0}^{-1}$ is used due to its proportionality to the conductivity and the imaginary part of the permittivity $\varepsilon^{\prime \prime }$ of the resonator medium [55]. Furthermore, instead of the resonant frequency, the frequency shift relative to a reference value—in this study, the signal for a clean filter—$\left|{\Delta f}_{\mathrm{res}}/f_{\mathrm{res},0}\right|$ was considered, which depends on the real part of the permittivity $\varepsilon^{\prime }$. Thus, both considered sensor signals are affected by different material properties.

## 3. Kinetics of Realistic Soot

As an example of how the GPF model can be used to support the sensor development, the influence of the reaction kinetics of different engine soot types was investigated. For this purpose, gasoline soot generated at four engine operating points, and PrintexU for comparison, which is often used as a soot representative, was analyzed regarding the kinetic parameters. These data were then used to simulate the sensor response during realistic driving cycles.

The gasoline soot was generated on an engine test bench equipped with a 1.8-liter, four-cylinder engine with gasoline direct injection. After flowing through a TWC, the particles of each operating point were deposited into a clean GPF without a catalytic coating. These filters were 5.2″ (132.1 mm) in diameter and 5″ (127 mm) in length with a cell density of 200 cpsi (channels per square inch). The filter walls themselves had a thickness $w_{s}$ of 8.5 mil (0.216 mm) with a porosity of 55% and a pore diameter of 13.5 µm. The four operating points differed in their load point, which corresponded to driving speeds of 120 km/h and 160 km/h, and in the air–fuel ratio *λ* at which the engine was operated. In addition to stoichiometric engine operation (*λ* = 1), these two load points were also driven with the standard specifications of the engine control unit under rich conditions (*λ* < 1). In particular, the air–fuel ratio was *λ* = 0.82 for the 120 km/h and *λ* = 0.90 for the 160 km/h load point. Furthermore, the injection timing and pressure were adjusted to ensure comparability with the *λ* = 1 operating points regarding the soot particle size. This led to an increased soot production, whereby faster loading of the particle filters could be accomplished. Thus, the costs for the examination of the sensor systems on engine test benches could be reduced. However, the comparability of this soot with soot from a standard engine operation has yet to be verified in further investigations.

The filters were loaded up to about 2 g/L soot per filter volume, which provided a sufficient amount of soot for the reaction kinetic analyses. Such a filter loading would also be possible in a real operation. Afterward, these soot-loaded samples were removed from their canning and mounting mat. The regeneration behavior was determined via thermogravimetric analysis (TGA) using a Seiko EXSTAR 6000 TG/DTA 6300 (Seiko Instruments Inc., Chiba, Japan). For the sample preparation, a slice was cut from the center of the GPF and coarsely crushed using a mortar. From the resulting powder, between 18 and 20 mg was placed in a ceramic crucible. PrintexU was also analyzed as comparative soot. For this purpose, a clean GPF was crushed and mixed with the quantity of synthetic soot corresponding to a load of 2 g/L (identified in the figures as “2 g/L PrintexU”). To determine the possible influence of the filter substrate on the regeneration behavior, pure PrintexU was also investigated using TGA. Due to the lower apparent density, only 3 mg could be placed in the crucible. The samples were heated at a constant rate of 5 K/min in a gas flow of 500 mL/min. To eliminate the influence of volatile components, the samples were heated to 900 °C in an initial step in a nitrogen atmosphere. Subsequent oxidation was performed by adding 2 vol.% oxygen during the entire heating period.

**Figure 4:**
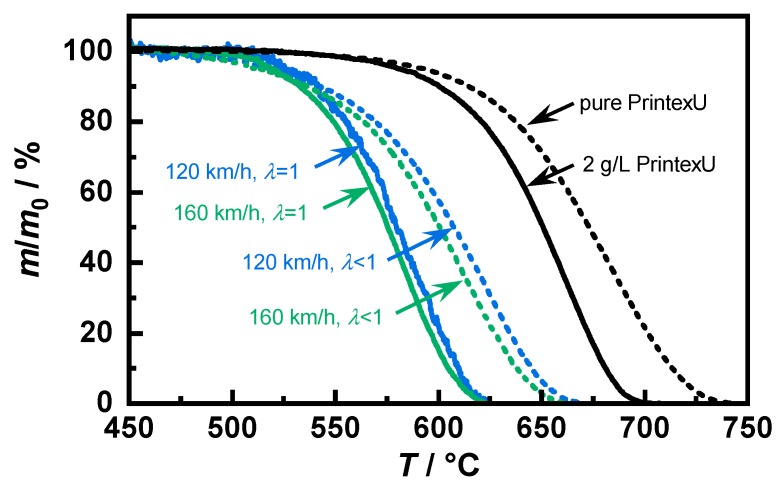
Soot oxidation measured using thermogravimetric analysis (TGA) with a heating rate of 5 K/min for different soot types.

The decrease in soot mass *m* relative to the total existing soot mass $m_{0}$ is plotted in Figure 4 as a function of temperature. The soot samples showed a quite different reaction behavior. Looking at the temperature at which 50% of the mass was converted, the stoichiometric operating points were about 75 °C below that of the PrintexU mixed with the substrate. Likewise, the samples produced under rich conditions also showed a slightly slower combustion temperature. In contrast, there were only minimal differences of less than 10 K between the two engine load points, whereby the “120 km/h” samples showed a slightly higher regeneration temperature independent of the air–fuel ratio. The comparison of both PrintexU samples shows that the pure soot combusted at higher temperatures. Therefore, future investigations and simulations should take into account the influence of the substrate material on the kinetic properties.

For an exact determination of the activation energy $E_{A}$ and the pre-exponential factor $k_{c,0}$, the conversion equation (Equation (15)) was transformed such that a separate consideration of both parameters was easily possible. By plotting the measured data from Figure 4 in the form of Equation (21) in an Arrhenius plot, a line was obtained for the oxidation following the assumed reaction, whereby in this study, a reaction order of *n* = 1 was used:

(21) ln(−∂∂tmm0·1cO2)−nln(mm0)=ln(kc,0Mc)−EART.

**Figure 5:**
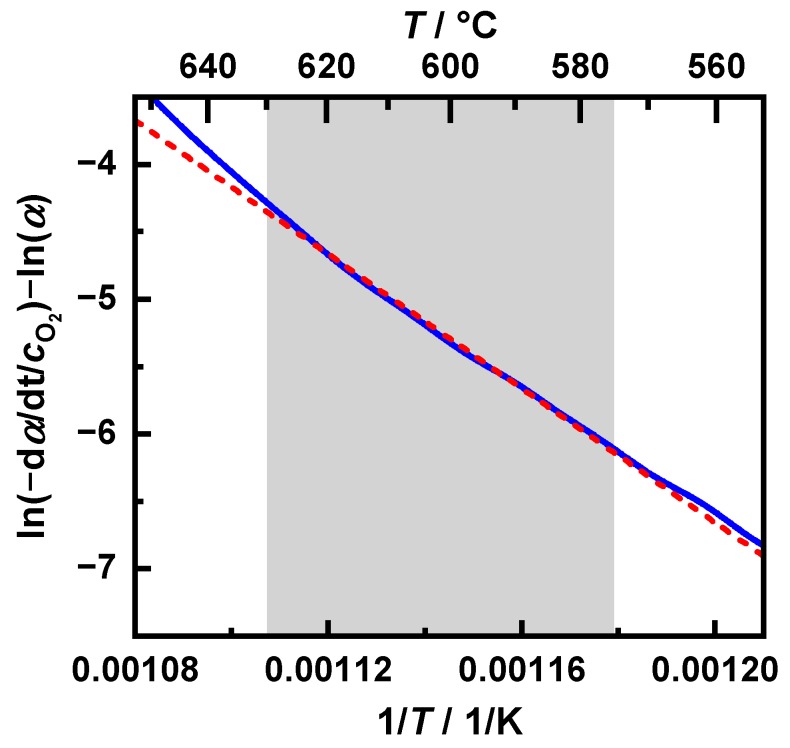
The oxidation rate of the “120 km/h, *λ* < 1” operation point of the temperature at a heating rate of 5 K/min in an Arrhenius plot with n = 1. The dotted red line corresponds to a linear fit in a range of 10% to 70% mass conversion (marked in grey), The slope correlates with the activation energy, while the *y*-axis intersection point is equal to the logarithm of the reaction rate.

A line was fitted to the linearized measurement data in the range from 10% to 70% mass conversion (from 0.9 *m*/*m*_0_ to 0.3 *m*/*m*_0_) to evaluate the parameters. The pre-exponential factor could then be determined using the *y*-axis intersection for this line, which corresponded to $\ln\left(k_{c,0}M_{c}\right)$. On the other hand, the negative slope was multiplied by the gas constant *R* to obtain the activation energy $E_{A}$. This is shown exemplarily for the operating point “120 km/h” in Figure 5. Here, as well as for the other investigated soot types, high linearity of the measured data was found.

The reaction parameters were determined using this method for all substrate–soot mixtures (Table 1). This confirmed the similarity of the kinetic parameters for operating points with identical air–fuel ratios.

**Figure 6:**
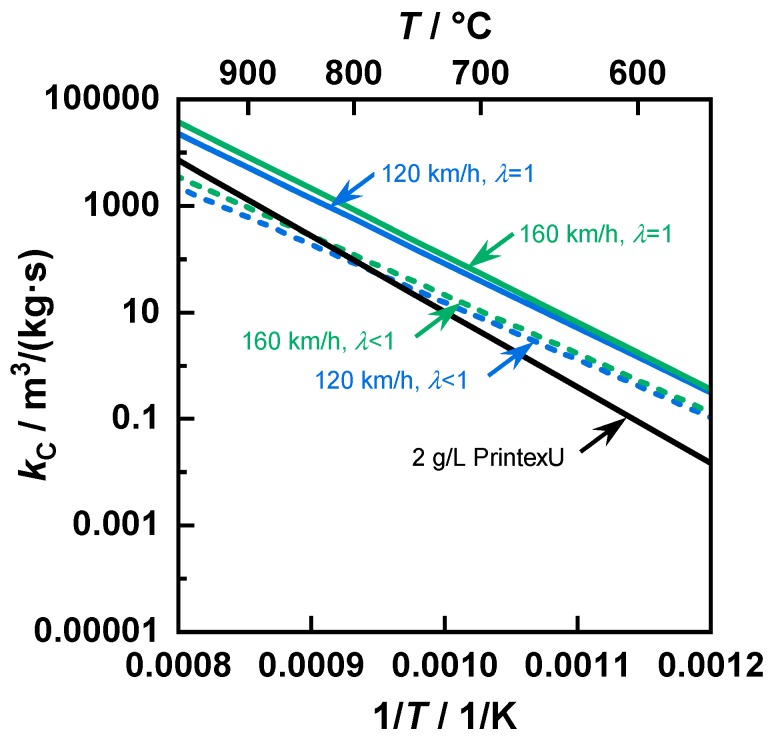
The reaction rate constant $k_{c}$ as a function of the inverse temperature for different soot types.

Regarding the reaction rate constant $k_{c}$, a similar temperature dependency was observed for the investigated gasoline samples at typical temperatures for GPFs (Figure 6). On the other hand, PrintexU had a steeper slope such that at temperatures above 800 °C, the kinetics was faster than for the samples produced in rich conditions. However, the reaction rates of the stoichiometric samples were not reached for typical exhaust gas temperatures. Furthermore, a comparison of the kinetics determined in this paper with reaction rates of different carbonaceous substances from the literature shows comparable temperature dependencies [52].

## 4. Application of the Model to Driving Cycles

**Figure 7:**
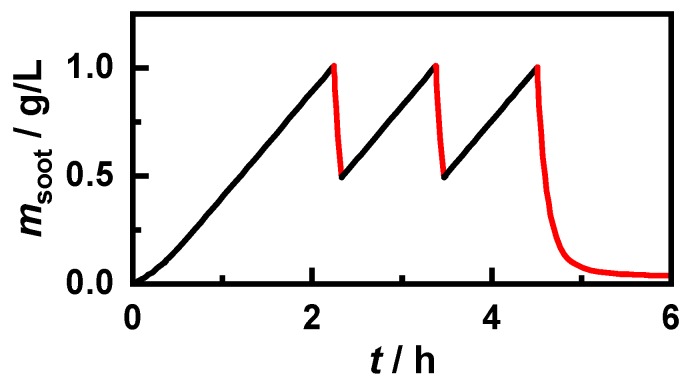
Stored soot mass with the “120 km/h, *λ* = 1” soot. After reaching 1 g/L, regeneration (marked in red) was initiated by adding 2 vol.% O_2_ until 0.5 g/L remained on the filter. After the third loading, permanent lean exhaust gas was present.

The above-determined reaction kinetic parameters were then considered in the model. In the following simulations, the parameters of the GPF used for the reaction kinetic investigations were applied. The permeability of the filter walls, the soot cake, and the soot deposited in the deep bed used in the simulations were determined by adapting the simulated differential pressure to engine test bench results. To simulate the effects of incomplete regenerations, a stationary engine operating point with a constant mass flow of 200 kg/h and a gas temperature of 600 °C at the inlet was assumed. This resulted in an average filter temperature $T_{\mathrm{GPF}}$ of 578 °C. However, in the outer areas of the filter, the temperature was slightly below 540 °C, whereby significant effects on the regeneration behavior were expected. The filter loading was achieved by adding a soot mass flow of 0.5 g/(L⋅h), relative to the filter volume. However, due to imperfect filtration efficiency, the storage rate in the GPF was slightly lower. Regeneration was initiated by adding 2 vol.% oxygen (which corresponded to an air–fuel ratio *λ* of approx. 1.1) as soon as a filter load of 1 g/L was reached. Such an increase of the oxygen concentration can occur in real operation, for example, by switching to a lean engine operation mode. No adjustments were made to the exhaust gas mass flow or exhaust gas temperature to facilitate the analysis of the effects caused by incomplete regenerations. Thus, a direct comparison of the simulated data with measurements on an engine test bench was consequently not possible. Once the soot load had fallen below 0.5 g/L, the regeneration was interrupted by reducing the oxygen concentration to zero. After three loadings, a complete regeneration was carried out. Due to the limited reaction rate, a small residual mass remained on the filter with gasoline soot, even after several hours in the lean exhaust gas. The course of the resulting soot mass is shown exemplarily for the “120 km/h, *λ* = 1” soot in Figure 7. In PrintexU-loaded filters, the mass increase only slowed down during the regeneration phases due to the much slower soot kinetics compared to gasoline soot. To be able to examine the effects of incomplete regenerations for PrintexU, the exhaust gas temperature was increased to 650 °C, which resulted in a mean GPF temperature of 625 °C.

**Figure 8:**
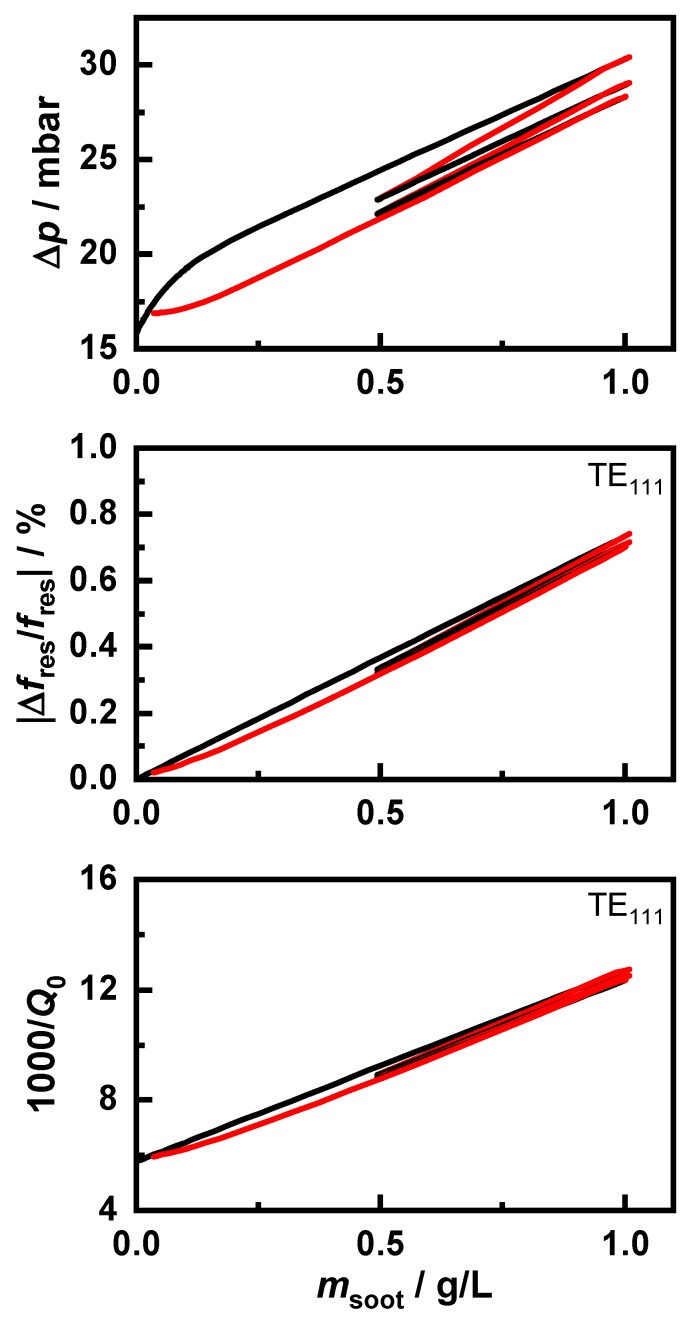
Sensor signals of the differential pressure sensor (Δ*p*) and the RF sensor ($\left|{\Delta f}_{\mathrm{res}}/f_{\mathrm{res},0}\right|$ and 1000/$Q_{0}$) as a function of the stored soot mass. The regeneration phases are marked in red.

**Figure 9:**
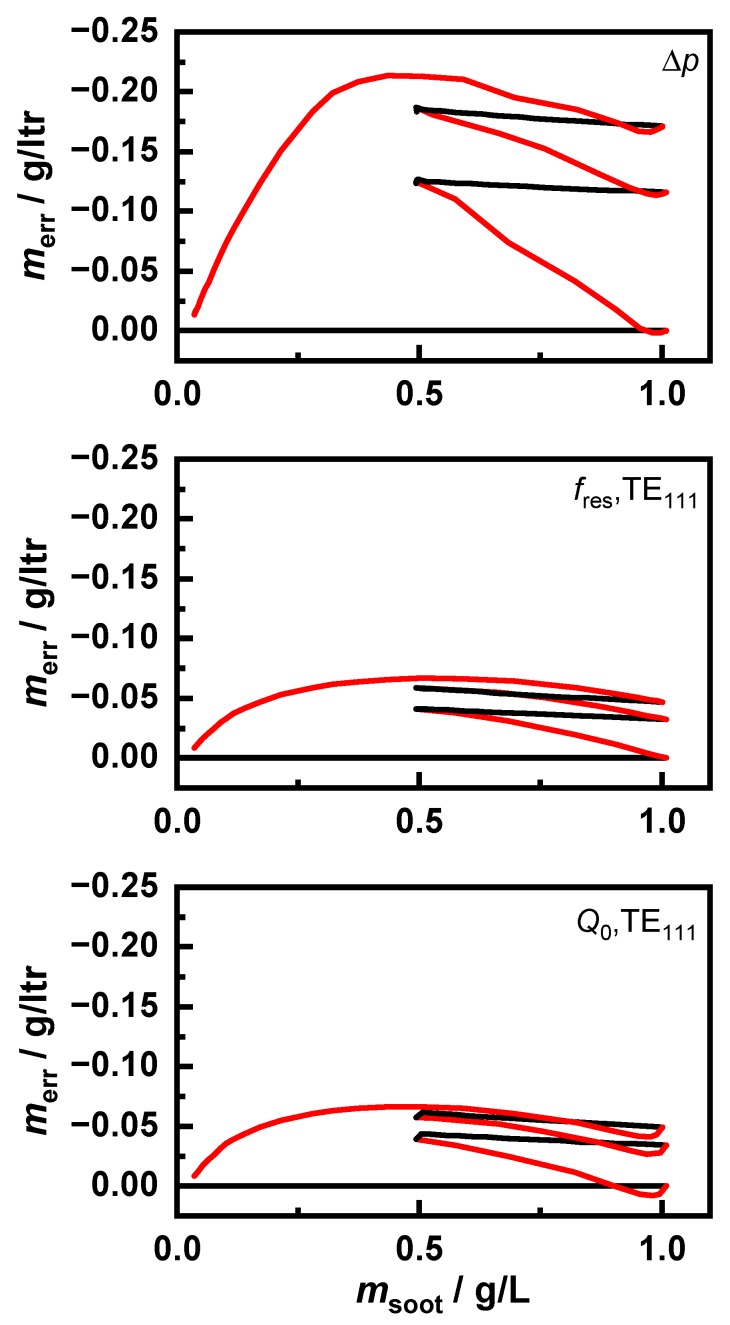
Sensor error in g/L as a function of the stored soot mass in the pressure differential sensor and the RF sensor based on $f_{\mathrm{res}}$ relative to $Q_{0}$. The regeneration phases are marked in red.

**Figure 10:**
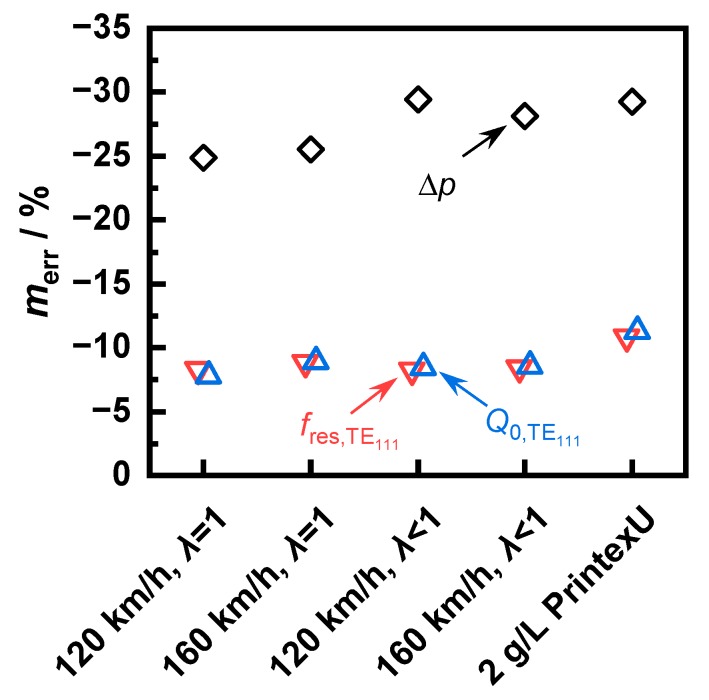
The proportional error of the Δ*p* and the RF sensors (evaluation of the resonant mode TE_111_) after the end of the first regeneration.

The resulting behavior of the differential pressure and the RF sensor is shown in Figure 8. For the sake of clarity, as with the sensor error in Figure 9, only the signal of the “120 km/h, *λ* = 1” signal is shown since the sensor signals of all soot types showed highly similar behavior. A detailed analysis of the differences between the soot types was made using the evaluation of the sensor error in Figure 10. The RF sensor could determine the soot mass using various signals. In this study, the frequency shift $\left|{\Delta f}_{\mathrm{res}}/f_{\mathrm{res},0}\right|$ and the change of the inverse quality factor ${\Delta Q}_{0}^{-1}$ of the resonant mode TE_111_ were considered.

Both the Δ*p* and RF sensors showed an almost linear behavior. With the differential pressure, the storage behavior in the deep bed at the beginning of the loading was clearly recognized due to the greater signal increase. Since soot burned simultaneously in the deep bed and the soot cake, a stronger decrease of the differential pressure was observed as only regeneration of the soot cake occurred. Additionally, soot, which burned very slowly in the colder regions of the GPF, affected the mass flow distribution through the filter due to the high flow resistance there. This also contributed to the sensor hysteresis during regeneration but to a smaller extent relative to the effect of the deep bed. Furthermore, during the following loading, this was counterbalanced by a changed storage behavior, whereas the backpressure loss due to the regeneration in the deep bed could not be compensated for due to the closed soot cake. In contrast, this did not influence the sensor signal of the RF sensor. However, this also showed a slight sensor inaccuracy due to incomplete regenerations independent of the considered resonant parameter.

To observe the deviations more closely, the mass determination error of the sensors is shown in Figure 9. To determine this sensor error, a known sensor characteristic was assumed based on the first loading of a clean filter. The simulated sensor signals were recalculated to a soot mass based on this characteristic. The sensor error $m_{\mathrm{err}}$ represents the deviation from the actual soot mass. This error was a systematic difference due to the changed soot distribution in the filter as a result of the incomplete regeneration and could also be observed in engine test bench measurements.

This clearly shows that the mass error was caused by the regenerations. However, with the subsequent loading steps, the error remained almost constant. Additionally, the increase of the error in the differential pressure signal was not constant but instead fluctuated slightly. The reasons for this should be clarified in further investigations. Furthermore, there are slight differences between the two signals of the RF sensor. The quality factor $Q_{0{,\mathrm{TE}}_{111}}$ detected a slightly higher soot mass at the beginning of the regeneration. However, at the end of the regeneration, as with the resonant frequency and the differential pressure, a too low soot mass was detected. This could be explained by the higher temperature sensitivity of $\varepsilon_{\mathrm{soot}}^{\prime \prime }$, which was relevant for the quality factor, compared to $\varepsilon_{\mathrm{soot}}^{\prime },$ as well as a local temperature increase due to the exothermic soot oxidation.

For better comparability of the minor differences between the operating points, the error related to the actual soot mass after the end of the first regeneration was investigated (Figure 10). At all operating points, the differential pressure sensor showed an error that was about three times higher than the error for both RF signals. In contrast, the differences between an evaluation of the resonant frequency $f_{{\mathrm{res},\mathrm{TE}}_{111}}$ and the quality factor $Q_{0{,\mathrm{TE}}_{111}}$ were marginal and each showed an error of less than 10%. Furthermore, there was a large variation in the differential pressure error between the soot types. A linear correlation with the reaction rate could not be found. Thus, the “120 km/h” soot in the stoichiometric case caused a smaller error than the “160 km/h” soot, whereas in rich conditions, it caused a larger error despite the lower reaction rate being constant in both cases.

More precise analyses of the causes of error for the differential pressure sensor can be made via a more detailed evaluation of the local soot mass distribution and the exhaust flow. An explanation for the error of the RF sensor could be found by taking a closer look at the field distribution in the cavity. The electromagnetic field became weaker toward the edges of the resonator, which corresponded with the cooler areas of the filter. Thus, the RF sensor showed a low sensitivity to soot located in these regions, where more soot remained after regeneration due to slower reaction kinetics. A possible way to reduce this error would be to change the field distribution in the filter, for example, by increasing the canning diameter while maintaining the GPF dimensions or by evaluating resonant modes at higher frequencies.

## 5. Conclusions and Outlook

For the further development of sensors, in particular for the RF sensor that is used for soot-loading detection of gasoline particulate filters, complex engine measurements are often necessary. To simplify this development work, a simulation model was developed in this study that precisely described the soot deposition and combustion processes using a two-dimensional examination of the GPF and the exhaust tract before and after these processes. Based on this, the resulting sensor signals from a differential pressure or radio-frequency measurement were determined and compared with each other. Using reaction kinetics analyses of gasoline soot at different engine operating points via thermogravimetric analysis, their oxidation behaviors were investigated in the model and their effect on the accuracy of both sensor concepts was shown. It was shown that the differential pressure sensor signals were subject to higher systematic errors during incomplete regenerations than the RF sensor signals. Additionally, the error was more dependent on the reaction kinetics. The reason for the error occurring with both sensor concepts was found in the changed soot distribution in the filter as a consequence of the soot oxidation.

To validate the simulation model in detail, various engine test bench measurements should be replicated in the future. For a more precise description of the RF sensor behavior, further investigations regarding the impact of different operating points on the dielectric properties of soot should be carried out in the future. Additionally, the model should be used to identify further influences on the sensor accuracy and help to develop methods for correcting the resulting errors.

## Figures and Tables

**Table 1:** Activation energy and pre-exponential factor of different soot types.

Soot Type	$E_{A}$ (kJ/mol)	$k_{c,0}$ (m^3^·kg^−1^·s^−1^)
120 km/h, *λ* = 1	232.6	$1.2\times 10^{14}$
160 km/h, *λ* = 1	239.8	$3.9\times 10^{14}$
120 km/h, *λ* < 1	206.7	$9.7\times 10^{11}$
160 km/h, *λ* < 1	211.7	$2.5\times 10^{12}$
2 g/L PrintexU	271.9	$1.7\times 10^{15}$

## Abbreviations

*a*	filter channel width
cpsi	cells per square inch
$c_{\mathrm{CO}}$	concentration of carbon monoxide
$c_{{\mathrm{CO}}_{2}}$	concentration of carbon dioxide
$c_{O_{2}}$	oxygen concentration in the exhaust gas
$c_{p,\mathrm{exhaust}}$	heat capacity of exhaust gas
$c_{\mathrm{soot}}$	soot concentration in the exhaust gas
DPF	diesel particulate filter
$d_{s}$	thickness of soot cake
$d_{s,\mathrm{deep}}$	virtual thickness of soot deposited in deep bed
$E_{A}$	activation energy for soot oxidation
*F*	factor equal to 28.454
$f_{\mathrm{CO}}$	factor describing the ratio of CO_2_ to CO during soot oxidation
$f_{\mathrm{res}}$	resonant frequency
GDI	gasoline direct injection
GPF	gasoline particulate filter
$k_{c}$	reaction rate constant
$k_{c,0}$	pre-exponential factor
$M_{c}$	molar mass of carbon
$m_{\mathrm{err}}$	sensor error
$m_{\mathrm{soot}}$	soot mass deposited in the filter
$m_{\mathrm{soot},\mathrm{bed}}$	soot mass collected using deep bed filtration
$m_{\mathrm{soot},\mathrm{cake}}$	soot mass deposited in the soot cake
${\dot{m}}_{\mathrm{exhaust}}$	exhaust gas mass flow rate
${\dot{m}}_{\mathrm{soot},\mathrm{in}}$	soot mass flow rate into the exhaust gas system
${\dot{m}}_{\mathrm{soot},\mathrm{exhaust}}$	soot mass flow through the filter walls
*n*	reaction order of the soot oxidation
$Q_{0}$	quality factor
${\dot{Q}}_{\mathrm{GPF}}$	heat flow into GPF caused by the exhaust gas
*R*	gas constant
RF	radio frequency
TE_111_	resonance mode in the filter canning
TGA	thermogravimetric analysis
$T_{\mathrm{GPF}}$	temperature of the GPF substrate
$T_{\mathrm{inlet}}$	gas temperature in the inlet channels
$T_{\mathrm{outlet}}$	gas temperature in the outlet channels
$v_{w}$	gas velocity through the filter channel walls
$w_{s}$	thickness of the filter channel walls
$\alpha_{\mathrm{cake}}$	filter penetrability caused by the soot cake
$\alpha_{\mathrm{deep}}$	filter penetrability caused by the deep bed filtration
$\alpha_{\mathrm{clean}}$	filter penetrability without the soot loading
$\alpha_{\mathrm{filter}}$	filter penetrability
Δp	differential pressure
$\varepsilon_{\mathrm{filter}}^{\prime }$	real part of the filter permittivity
$\varepsilon_{\mathrm{filter}}^{\prime \prime }$	imaginary part of the filter permittivity
$\varepsilon_{\mathrm{soot}}^{\prime }$	real part of the soot permittivity
$\varepsilon_{\mathrm{soot}}^{\prime \prime }$	imaginary part of the soot permittivity
$\varepsilon_{\mathrm{substrate}}^{\prime }$	real part of the filter substrate permittivity
$\varepsilon_{\mathrm{substrate}}^{\prime \prime }$	imaginary part of the filter substrate permittivity
η	dynamic viscosity of the exhaust gas
$\eta_{\mathrm{bed}}$	deep bed filtration efficiency
$\eta_{\mathrm{cake}}$	soot cake filtration efficiency
$\kappa_{0}$	filter wall permeability
$\kappa_{\mathrm{channel}}$	filter channel permeability
$\kappa_{s}$	soot cake permeability
$\kappa_{s,\mathrm{deep}}$	deep bed permeability
*λ*	air–fuel ratio
ρ	exhaust gas density
$\rho_{\mathrm{GPF}}$	filter density
$\rho_{\mathrm{soot}}$	soot density
$\rho_{\mathrm{substrate}}$	filter substrate density
σ	conductivity
ϕ	fraction of the soot in the deep bed contributing to the soot cake formation
μ	magnetic permeability
